# Infectious disease surveillance for refugees at borders and in destination countries: a scoping review

**DOI:** 10.1186/s12889-022-12646-7

**Published:** 2022-02-04

**Authors:** Majd Saleh, Zeina Farah, Natasha Howard

**Affiliations:** 1 Department of Global Health and Development, London School of Hygiene and Tropical Medicine, 15-17 Tavistock Place, London, WC1H 9SH UK; 2 Epidemiological Surveillance Program, Ministry of Public Health, Beirut, Lebanon; 3 Saw Swee Hock School of Public Health, National University of Singapore and National University Health System, 12 Science Drive 2, Singapore, 117549 Singapore

**Keywords:** Refugees, Migrants, Surveillance, Infectious diseases, Policies, Protocols

## Abstract

**Background:**

Data on infectious disease surveillance for migrants on arrival and in destination countries are limited, despite global migration increases, and more are needed to inform national surveillance policies. Our study aimed to examine the scope of existing literature including existing infectious disease surveillance activities, surveillance methods used, surveillance policies or protocols, and potential lessons reported.

**Methods:**

Using Arksey and O’Malley’s six-stage approach, we screened four scientific databases systematically and 11 websites, Google, and Google Scholar purposively using search terms related to ‘refugee’ and ‘infectious disease surveillance’ with no restrictions on time-period or country. Title/abstracts and full texts were screened against eligibility criteria and extracted data were synthesised thematically.

**Results:**

We included 20 eligible sources of 728 identified. Reporting countries were primarily European and all were published between 1999 and 2019. Surveillance methods included 9 sources on syndromic surveillance, 2 on Early Warning and Response (EWAR), 1 on cross-border surveillance, and 1 on GeoSentinel clinic surveillance. Only 7 sources mentioned existing surveillance protocols and communication with reporting sites, while policies around surveillance were almost non-existent. Eleven included achievements such as improved partner collaboration, while 6 reported the lack of systematic approaches to surveillance.

**Conclusion:**

This study identified minimal literature on infectious disease surveillance for migrants in transit and destination countries. We found significant gaps geographically and on surveillance policies and protocols. Countries receiving refugees could document and share disease surveillance methods and findings to fill these gaps and support other countries in improving disease surveillance.

## Background

Disease surveillance contributes to epidemic control and other important public health responses [1–3]. The World Health Organization (WHO) defines disease surveillance as “ongoing systematic collection, analysis, and interpretation of outcome-specific data for use in planning, implementing and evaluating public health policies and practices” [4]. Surveillance differs from medical screening, the aim of which is clinical, as surveillance is broader and involves analysing health issues for disease interventions and prevention [4–6]. Infectious disease surveillance provides ongoing information on the health status of a population, which contributes to morbidity and mortality prevention, improves health service provision, and guides population health programmes [4, 7, 8].

Surveying and quantifying the healthcare needs of displaced populations, especially newly arrived ones, is important for disease prevention and resource allocation for often vulnerable people [9, 10]. Infectious disease surveillance during humanitarian crises can enable ongoing information for action, especially in displacement settlements [4, 7, 8]. Both formal (e.g. traditional ‘refugee camps’) and informal displacement settlements can be overcrowded and lack basic needs, e.g. water, sanitary supplies, nutritious food, and environmental protection [4, 7, 8]. Infectious disease concerns among refugees arriving in destination countries are diverse, with a recent review among asylum-seekers and refugees in Europe for 2010–2016 finding tuberculosis and Hepatitis B most prevalent with malaria, Hepatitis C, cutaneous diphtheria, louse-born relapsing fever, and shigellosis also common [11]. The United Nations Refugee Agency (UNHCR) reported that 75% of outbreaks in its refugee camps in 2009–2017 were due to measles, cholera, and meningitis [12]. Hence, disease surveillance is required for identifying outbreaks and initiating timely interventions.

From 2011, Europe experienced significantly increased forced migration from Syria along with other war-devastated countries in the Eastern Mediterranean, South Asia, and North Africa regions [13–15]. Consequently, the European Centres for Disease Control (ECDC) encouraged establishment of syndromic surveillance for refugee populations within routine national surveillance systems [13, 14]. Syndromic surveillance entails early detection of possible disease outbreaks by using clinical rather than laboratory confirmed diagnoses to enable faster responses that can reduce morbidity and mortality [14, 16]. In 2016, ECDC published a three-phase guidance (i.e. preparatory, pilot, implementation) on establishing syndromic surveillance systems for countries receiving refugees [13, 14]. This helped countries such as Germany, Greece, Italy, and Spain, develop syndromic surveillance to respond more quickly to migrants taking the Mediterranean route [15, 17–21].

We aimed to identify and summarize the literature related to infectious diseases surveillance targeting refugees at borders or in destination countries. Objectives were to: (i) identify national, international, and cross-border infectious diseases surveillance activities targeting refugees; (ii) examine how these surveillance activities were conducted, including protocols, regulations, and policies developed in relation to surveillance activities; and (iii) synthesise any major achievements or challenges identified.

## Methods

### Study design

We conducted a scoping review, using Arksey and O’Malley’s six-stage framework with Levac et al’s revisions, as detailed in Woodward et al. [22]. These stages are: (i) defining the research question; (ii) identifying documentary sources addressing the research question; (iii) selecting sources that meet inclusion criteria; (iv) charting/extracting relevant data; (v) synthesising and analysing data; and (iv) consulting topic experts to identify additional sources or sense-check initial findings as appropriate. We selected a scoping design anticipating that our literature would be limited to relatively few sources and heterogeneous in design, type, methods, focus, and quality.

### Defining the research question

Our research question was: “What is the scope and nature of the literature on infectious disease surveillance for refugees, as defined either by mandate or 1951 Convention, including surveillance methods, policies/protocols, and lessons learned?”

### Identifying sources

The lead author searched four databases systematically (i.e. EMBASE, Global Health, PubMed, Jstor), 11 selected websites purposively (i.e. International Organization for Migration [IOM], Eurosurveillance, World Health Organization [WHO], including six regional office sites, bulletins, weekly epidemiological record), and Google and Google Scholar purposively for the first 100 hits. Purposive searching, as used for websites and search engines, is equivalent to non-probability sampling in that researchers rely on personal judgment to rapidly prioritise sources from a broad range of settings that are as relevant as possible to the review and contain sufficiently rich data [23].

Table 1 shows study definitions used. The search strategy used terms related to” refugee” and” infectious disease surveillance” [24, 26]. Our study focused on surveillance as opposed to screening [6], as the aim of the latter is primarily clinical and involves detecting specific diseases within a high-risk population not yet symptomatic [5, 6]. Screening is a short-term activity performed at different intervals, while surveillance is broader, continuous, and involves analysing health issues for disease interventions and prevention [4–6]. However, we included screening in our search terms, as they are sometimes used interchangeably, and manually excluded documents only addressing screening. Similarly, though our study focused on mandated refugees, we included terms related to ‘migrant,’ ‘displaced,’ and ‘asylum-seeker’ populations, so as not to miss relevant documents that used different terms for people we defined as refugees, and manually excluded ineligible documents. Search terms were consistent, with subheading and MESH terms revised as needed. For example, in Ovid EMBASE the search used: (disease surveillance OR screening [subheadings]) AND (migrant OR refugee OR “asylum seeker” OR “displaced” [search terms]) AND (syndromic OR infectious OR infection OR “communicable disease” [subheading]) AND (policy OR “organizational policy” OR “public policy” [subheadings] OR protocol [search term]).

**Table 1:** Study definitions

Asylum-seeker	“An individual who is seeking international protection. In countries with individualized procedures, an asylum seeker is someone whose claim has not yet been finally decided on by the country in which he or she has submitted it. Not every asylum seeker will ultimately be recognized as a refugee, but every recognized refugee is initially an asylum seeker” [24]
Destination country	“In the migration context, a country that is the destination for a person or a group of persons, irrespective of whether they migrate regularly or irregularly” [24]
Displacement	“The movement of persons who have been forced or obliged to flee or to leave their homes or places of habitual residence, in particular as a result of or in order to avoid the effects of armed conflict, situations of generalized violence, violations of human rights or natural or human-made disasters” [24]
Displacement settlements	Used here as an umbrella term to refer to all forms of displaced communal living, including formal displacement camps, informal/irregular settlements, emergency accommodation, reception and detention centres, but not including open/dispersed
Irregular/ undocumented migration	“Movement of persons that takes place outside the laws, regulations, or international agreements governing the entry into or exit from the State of origin, transit or destination” [24]
Forced displacement	Involuntary or coerced movement of a person or people away from their home or home region, e.g. “as a result of persecution, conflict, generalized violence or human rights violations” [25]
Migrant	“An umbrella term, not defined under international law, reflecting the common lay understanding of a person who moves away from his or her place of usual residence, whether within a country or across an international border, temporarily or permanently, and for a variety of reasons” [24]
Refugee (mandate)	“A person who qualifies for the protection of the United Nations provided by UNHCR, in accordance with UNHCR’s Statute and, notably, subsequent General Assembly’s resolutions clarifying the scope of UNHCR’s competency, regardless of whether or not he or she is in a country that is a party to the 1951 Convention or the 1967 Protocol – or a relevant regional refugee instrument – or whether or not he or she has been recognized by his or her host country as a refugee under either of these instruments” [24]
Refugee (1951 Convention)	“A person who, owing to a well-founded fear of persecution for reasons of race, religion, nationality, membership of a particular social group or political opinion, is outside the country of his nationality and is unable or, owing to such fear, is unwilling to avail himself of the protection of that country; or who, not having a nationality and being outside the country of his former habitual residence as a result of such events, is unable or, owing to such fear, is unwilling to return to it” [24, 26]
Surveillance	“ongoing systematic collection, analysis, and interpretation of outcome-specific data for use in planning, implementing and evaluating public health policies and practices” [4]
Syndromic surveillance	“Syndromic surveillance can be defined as a form of early detection of possible outbreaks by using symptoms prior to any laboratory diagnosis” [14, 16]
Transit country	“In the migration context, the country through which a person or a group of persons pass on any journey to the country of destination or from the country of destination to the country of origin or of habitual residence” [24]
Vulnerability	“Within a migration context, vulnerability is the limited capacity to avoid, resist, cope with, or recover from harm. This limited capacity is the result of the unique interaction of individual, household, community, and structural characteristics and conditions” [24]

### Selecting sources

We agreed on eligibility criteria iteratively, from initial criteria based on the research question and research data sources (Table 2). The lead author removed duplicates using EndNote software and screened titles and abstracts, then remaining full texts, against eligibility criteria in discussion with co-authors. Issues were resolved by co-author consensus.

**Table 2:** Eligibility criteria

Criteria	Include	Exclude
1. Context	Any country experiencing in-migration.	No migration mentioned.
2. Topic	Mention of disease surveillance targeting refugees, as defined either by mandate or 1951 Convention.	Target populations include only one group of migrants who are not necessarily refugees (e.g. only asylum-seekers, only foreign-born persons, immigration detainees, labour migrants, internally-displaced).
3. Outcomes	Processes and methods of infectious disease surveillance for refugees: a. organizational aspects of surveillance; b. policies or protocols related to surveillance; c. assessment outcomes of surveillance activities.	Only describes clinical screening, ecological surveillance, forecasting studies, prospective one-time studies, cross-sectional or epidemiological studies to find prevalence of diseases, access to healthcare, ethical considerations and human rights aspects of surveillance, non-communicable diseases surveillance, prevention activities of infectious diseases, mental health studies, TB or HIV screening.
4. Source type	All journal article types (e.g. research, commentary, review).	4. Duplicates, e.g. conference abstracts for which an article exists.
5. Time-period	Any	NA
6. Language	Any language with an English abstract available.	No English abstract.

### Extracting data

We extracted data from each source to an Excel file under the following headings: lead author, publication year, target population, study methods, surveillance system description, surveillance methods used, protocols and policies used, and lessons learned.

### Synthesis

We collated descriptive data on included sources quantitatively (i.e. publication year, source type, countries included, primary methods, target populations described). We synthesised outcome data thematically on surveillance systems, methods, protocols/policies, and lessons learned, using a deductive approach described by Braun et al. [27].

### Consulting stakeholders

The lead author presented initial findings at the XII International Epidemiological Association-Eastern Mediterranean Region Scientific Meeting in Beirut in 2019. Ten experts on surveillance and refugee health provided feedback during the presentation or informally afterwards, which the lead author recorded using a notebook and used to strengthen this review.

## Results

### Scope and nature of documents

**Fig. 1:**
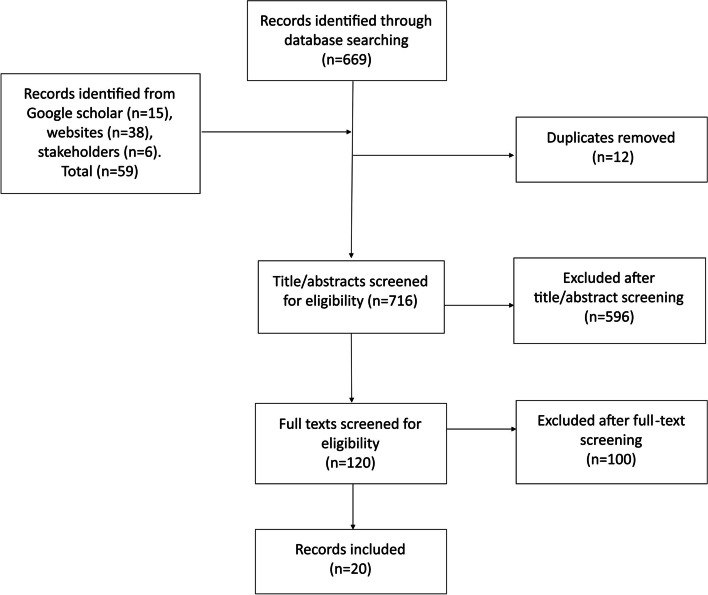
PRISMA flow diagram

Figure 1 provides the PRISMA flow diagram for 20 sources included of 728 identified through searches. Publication dates began with one source in 1999 (5%) and increased somewhat to a peak of 4 in 2018 (21%), only one in 2019, and none in 2020. Twelve (60%) were journal articles, five (25%) were evaluation or technical reports, two (10%) were WHO updates on EWAR, and one (5%) was a letter to editors.

Countries included were diverse, but predominantly European (i.e. 13 sources), 4 for Italy, 2 for Greece, 1 each for Albania, Germany, Macedonia, and Spain, while 1 included six European countries, and 2 discussed Europe as a region [17–21, 28–35]. Three from the Africa region discussed camp-based surveillance in Cote d’Ivoire and Sudan, disputed Sudan-Chad borders, and Minawao in Cameroon [36–38]. Two from the Asia region discussed surveillance in Bangladesh and the Myanmar-Thailand border [39, 40]. One from the Americas region discussed binational surveillance on the United States (US)-Mexico border [41]. One source was global, including 24 countries [42].

Terminology was also diverse. Only 8 sources used the term ‘refugee’ in describing target populations [30, 33–35, 37–40], 8 used ‘migrant’ as a general term [15, 17, 19–21, 32, 41, 42], and 4 combined terms, describing ‘migrants and refugees’, ‘displaced and refugees’, ‘refugees and asylum-seekers’ as the population of interest [18, 28, 29, 36]. Most sources (13; 65%) were descriptive rather than analytical, while 4 conducted health system assessments [19–21, 37], 1 used scoping methods [28], 1 used descriptive epidemiological statistics [18], and 1 provided authors’ opinions [30].

### Thematic synthesis

We synthesised outcomes under four deductive themes: (i) infectious disease surveillance targeting refugees and migrants; (ii) surveillance methods used; (iii) protocols and policies used; and (iv) reported lessons and limitations described. Table 3 summarises findings by source and theme.

**Table 3:** Sources by year published and outcomes by theme

Lead author, year	Target population	Type (topic)	Surveillance system	Surveillance methods	Protocols/policies	Lessons
Valenciano et al., 1999	Kosovar refugees in Albania	Descriptive (surveillance activities)	Emergency surveillance system	Refugee camps were assigned a medical agency, with weekly reporting forms sent by car or fax, including null forms for specific high risk diseases. IPH prepared weekly updates for dissemination.	IPH Albania provided reporting sites with lists of diseases and case definitions for easier identification and reporting.	Very sensitive system led to some false alarms, but was acceptable for medical staff as it was simple and led to close collaboration between partners.
Brusin, 2000	Kosovar refugees in Macedonia	Descriptive (surveillance activities)	Surveillance system targeting refugee camps	The surveillance system was divided into: (i) 14 immediate and weekly reportable diseases in a shared reporting form, via telephone or FM radio to both Republic Institute for Health Protection (RIHP) and WHO; (ii) other diseases. WHO compiled reports and published a weekly bulletin.	Protocols were formulated and sent to all health facilities working in the refugee camps. These protocols included case definitions, forms, important contacts, as well as instructions on how, who, and when the form is to be filled	
WHO, 2004	Displaced and refugee populations in Sudan and Chad	Descriptive	WHO early warning surveillance	WHO monitored 12 infectious diseases and other health events.		Effective as a real-time reporting system with outbreaks easily detectable.
Kouadio et al., 2009	Liberian Refugees in Cote d’Ivoire transit camps	Descriptive	Diseases Surveillance Team (DST) for surveillance and outbreak investigation	Under DST, active surveillance by medical staff, e.g. door-to-door searches, and passively by camp health units, and results disseminated through routine meetings.	Advisory team to support camp-level decision-making. Mandatory dissemination meetings.	Described as “active and comprehensive,” helped reduce transmission in camps due to involvement of community members.
Waterman et al., 2009	Travelers crossing US-Mexico border	Descriptive	Collaborative surveillance including migrants	In 1997, due to frequent infectious diseases at US-Mexico border, binational surveillance was established using common case definitions. To maintain communication, US-CDC conducts annual binational meetings on border infectious disease surveillance.	Guidelines drafted, as per WHO IHR requirements, for communicating epidemiological data across borders.	Surveillance coordination was successful and led to an agreement between Canada, US, and Mexico to strengthen collaboration.
Riccardo et al., 2011	North African migrant centres in Italy	Descriptive	Syndromic surveillance	Surveillance defined and implemented 13 syndromes for migration centres, with cases reported daily via fax that fit case definitions. CNESPS-ISS analysed data, monitored alarms, and published weekly surveillance updates online.	An official guidance document for migration centres was sent to respective regions.	This surveillance was the main source of migrant data and enabled detection of potential outbreaks, though challenges included some centres not following protocols.
McCarthy et al., 2013	Migrants attending clinics in 24 countries	Descriptive	GeoSentinel surveillance of clinic sites targeting migrants	GeoSentinel surveillance in 24 countries across 6 continents to detect infectious diseases among migrants, mainly in travel and tropical medicine clinics. Cases are reported if they have crossed international borders.	A data collection protocol was developed and reviewed by US-CDC’s institutional review board.	Gives insight on main infectious diagnoses and demographics of migrants.
Turner et al., 2013	Myanmar refugees in Maela camp in Thailand on Myanmar border	Descriptive	Enhanced respiratory virus surveillance in camp hospital	Hospital-based surveillance, 2009–2011, to identify Pneumonia virus burden among Myanmar refugees in Northwest Thailand. Laboratory-enhanced respiratory surveillance involved trained personnel actively searching for pneumonia diagnoses in in-patient department admission logs. Those who met case definitions and had agreed were further investigated including RT-PCR testing.		Information generated from enhanced surveillance enabled identification of pneumonia aetiology in the camp population.
Napoli et al., 2014	Migrants arriving in Italy	Descriptive	Ad hoc early warning system-syndromic surveillance	Paper-based forms sent to MOH and CNES by email or fax. Once received, data were entered on an electronic database and an aggregated report published online and disseminated to reporting sites and MOH officials.	Centres received a detailed protocol listing syndromes to report and case definitions. They want to allow reporting centres to apply legally existing surveillance systems	System limitations meant it cannot be sustained, which needs to be addressed.
WHO-Euro, 2014	Sicily, Italy	Assessment (information component of health system capacity to handle migrant influx)	Previous emergency syndromic surveillance	Established in 2011, the number of reporting sites has dropped and sites do not collect information in a systematic way		System not systematic. Health-workers in migrant centres mentioned some parts of the syndromic surveillance system needed to be made clearer.
Germinario et al., 2015	Refugees in Apulian Asylum-Seeker Centres (CARA)	Descriptive. Was voluntary, hence participants chose to participate.	Multiple types of surveillance	Many activities, e.g. polio circulation surveillance, TB screening, Seroprevalence of viral hepatitis and HIV, and immunization programme. Syndromic surveillance is described as the first to apply this surveillance. Methods used are the same as described in other Italy publications, including notification of 13 syndromes to authorities via fax.	An available protocol was mentioned, informing centres how to report.	Presence of syndromic surveillance showed communicable disease in crowded places as was the case in centres. Highlighted the need for EU regulations on communicable diseases screening among migrants.
Riccardo et al., 2015	Refugee in EU	Journal letter to editor	Limitations and lack of a common surveillance approach			Information in the European surveillance database include place of birth and nationality, but a review showed limited information on: legal status of migrants, country of origin or route taken, and time of arrival. This makes it hard to stratify migrant groups.
WHO-Euro, 2015	Greece	Assessment (information component of health system capacity to handle migrant influx)	No systematic syndromic surveillance system	Migrant data collected by organizations offering health services at refugee reception centres. Data collection was non-systematic with no central system linking it together		No central surveillance system, different databases maintained by different partners. WHO recommended that these be linked for a unified system
Riccardo, 2017	Refugees and migrants in EU	Evaluation	Common Approach for Refugees syndromic surveillance simulation	A preparedness exercise in phases: 1. Preparatory: Italy assisted partner countries to set up surveillance. 2. Pilot/simulation: tested plans developed in preparatory phase. 3. Monitoring and evaluation of these surveillance systems focused on completeness, timeliness, simplicity, and acceptability attributes.	Protocols, procedures and tools were developed in the preparatory phase, leading to “procedures for syndromic surveillance in migrant reception/detention facilities” which provides case definitions, statistical testing, and an online platform with a section on trainings and simulation.	
HCDCP, 2017	Refugees/migrants arriving in Greece	Epidemiological report	Point-of-care syndromic surveillance	Reported 14 predefined syndromes or health conditions found at arrival centres, daily reporting to KEELPNO to analyse data nationally and for each centre separately.		Some diseases caught through mandatory notification system “that operates in parallel with the surveillance system in Points of Care for refugees/migrant”
Bozorgmehr et al., 2018	Refugees and asylum-seekers in EU	Scoping study	Surveillance activities targeting refugees and asylum-seekers	Collecting data and notification of diseases were the responsibility of national institutions in 18 of 27 representatives interviewed, who were also responsible for data collection for refugees and asylum-seekers. Established notification system required data to be transferred from regional to national levels.	Interviewees described data collection guidance and methods for data recording as insufficient.	Due to the burden of the ‘refugee crisis’, ad hoc measures needed to be taken.
Sarma et al., 2018	Migrants in mass accommodation in Germany	Descriptive (tool for syndromic surveillance)	Syndromic surveillance	Reporting 13 syndromes within 24 h to the national public health institute, i.e. Robert Koch-institute (RKI) surveillance team. Aggregate numbers were sent online, via telephone, or fax. RKI analysed data and conducted regular meetings between reporting sites and the surveillance team.	Surveillance team developed a toolkit, hosted on the RKI website, with information on data collection including sheets, Excel worksheet for analysis, and other supporting documents	The system was flexible with timely reporting and an obvious improvement in public health interventions, though data transfer by fax or online was considered inefficient.
WHO-SEARO, 2018	Rohingya refugees in Cox’s Bazar	Descriptive	WHO early warning surveillance	Reporting from 155 health facilities in camps for follow-up and analysis by WHO epidemiologists.		Effective as a real-time reporting system, with outbreaks easily detectable.
WHO-Euro, 2018	Spain	Assessment (information component of health system capacity to handle migrant influx)	Syndromic surveillance	Syndromic surveillance conducted through health-worker reporting of migrant medical check-ups to a national disease registry.	No specific protocol for early warning and response.	“While syndromic surveillance is carried out by health staff during individual medical assessments of migrants, communication with the public health authorities needs to be ensured.”
Amabo et al., 2019	Minawao refugee camp, Cameroon	Description and evaluation of diarrheal surveillance system	Diarrheal disease surveillance system	Surveillance supervised by Mokolo Health District Office in camps directed by non-governmental organizations, International Medical Corps (IMC), and Médecins Sans Frontières (MSF). The diseases surveyed all diarrheal in nature. Notifications were from both passive and active reporting, on a weekly basis. Surveillance officers aggregated data from sites into a reporting sheet. Using a hierarchical reporting system, the data ends up at the Ministry of health (MoH) where reports are shared with stakeholders.	For the assessment of the surveillance system, they used the protocols set by the US Centers for Disease Control and Prevention (CDC). For surveillance activities, the camp surveillance system used the case definitions set by the National IDSR guidelines from MoH Cameroon	They found that the surveillance system was successful in identifying interventions for outbreaks such as developing a committee and vaccination coverages. They also found that the tools used were standardized.

### Infectious disease surveillance targeting refugees and migrants

Most sources described migrant disease surveillance in Europe (13; 65%). In initial sources, published in 1999 and 2000, Valenciano et al and Brusin described syndromic disease surveillance systems established in two bordering countries, Albania and Macedonia, in response to the Kosovo crisis [34, 35].

After 2011, specific syndromic surveillance systems were developed in European countries to address increased migrant numbers more quickly. Three examples from Italy described disease surveillance for refugees, primarily from North Africa [15, 32, 33]. One described 6 months of syndromic surveillance in migration centres [32], results of 2 years of syndromic surveillance operated in parallel with existing routine statutory surveillance [15], and multiple other types of surveillance [33]. Riccardo et al. presented surveillance problems during migrant arrivals in the European Union (EU) in a letter to Eurosurveillance journal [30] and described the Common Approach for Refugees (CARE) Syndromic surveillance simulation implemented as a preparedness exercise in Italy [29].

WHO European Regional Office (EURO) assessed health system capacities of several migrant-receiving countries, including in Italy [19], Spain [20], and Greece [21]. The Italy assessment reported that Sicily had a syndromic surveillance system for migrants since 2011, while for the rest of Italy this appeared less active [19]. The Greece assessment suggested regular surveillance activities but no formal system established [21], while migrant disease surveillance was mentioned for Spain with no further details provided [20]. Syndromic surveillance systems were also documented in Greece [18] and Germany [17]. A scoping study, including key informant interviews in six European Union (EU) countries, described surveillance targeting refugees and asylum-seekers [28].

Two Asia region sources described a three-year enhanced hospital-based respiratory virus surveillance programme in a Myanmar refugee camp in Northwest Thailand, to examine pneumonia burden among migrants living on the border [40], and surveillance in Rohingya refugee camps in Cox’s Bazar Bangladesh (WHO-SEARO, 2018).

Two Africa region sources described Early Warning and Response (EWAR) networks in Darfur, Sudan, and Chad for displaced and refugee populations [36] and surveillance in Liberian refugee transit camps in Cote d’Ivoire [38]. One from Cameroon described activities of a diarrheal disease surveillance in Minawao refugee camp for an evaluation of the system [37].

One Americas region source described collaborative surveillance activities on the USA-Mexico border [41]. One global source briefly describes infectious disease surveillance activities in GeoSentinel clinic sites targeting migrants in 24 countries across six continents [42].

### Surveillance methods

Five sources reported surveillance activities supervised or implemented by national public health institutes such as the Institute of Public Health in in Albania, the National Centre for Epidemiology and National Institute of Health in Italy, and Robert Koch Institute in Germany [15, 17, 20, 34, 35]. A few reported assistance or implementation of surveillance systems by international organizations such as WHO in Bangladesh, Darfur, Sudan and Chad, UNHCR and WHO in Macedonia, and US Centres for Disease Control (US-CDC) on the US-Mexico border [34–36, 39, 41].

Notifiable disease lists were mentioned for 7 countries, usually consisting of 12–14 diseases and syndromes that were similar across countries [17, 18, 32, 34–36]. For example, all lists included acute respiratory infections, meningitis, and diarrhoea. Others included nationally relevant diseases, such as malaria in Greece, Sudan, and Chad, or non-infectious concerns such as psychological and cardiovascular diseases in Albania [18, 34, 36]. Four mentioned the provision of case definitions along with the list [15, 17, 32, 34].

Reporting approaches were passive or active. For passive reporting, as documented in Albania, Macedonia, Italy, Germany, and Cote d’Ivoire transit camps, reports from camps or immigration centres were sent to reporting authorities via online database, fax, email, telephone, radio, or vehicle [15, 17, 34, 35, 38]. Active case finding, as documented in Albania, Cote d’Ivoire transit camps, Cameroon, and Thailand, included door-to-door searches and reviews of medical registers [34, 37, 38, 40]. Reporting speed was either immediate, if fitting immediate notifiable criteria as in Macedonia [35]; daily as in Italian, German, and Greek syndromic surveillance systems [17, 18, 32]; or weekly as in Albania and Cameroon [34, 37].

Coordination meetings between reporting authorities, surveillance teams, reporting sites, and stakeholders were conducted either daily as in Cote D’Ivoire transit camps, weekly as in Macedonia, or monthly as in Germany [17, 35, 38]. One source reported annual binational meetings for border surveillance between US and Mexico authorities [41]. Dissemination of information, as statistical reports or bulletins, was most often weekly and shared during coordination meetings, on national surveillance program websites, via email, or as hard copies [15, 17, 32, 34, 35, 38].

All except four sources described national surveillance systems that tracked migrants. Exceptions were subnational surveillance in Apulia Italy [33] and border areas of Myanmar [40]. Additionally, Waterman et al described binational cross-border surveillance collaboration, with common case definitions established [41]. Finally, McCarthy et al described global GeoSentinel surveillance, mainly through specialised travel and tropical medicine clinics [42].

### Surveillance protocols, guidelines, and policies

Eight sources mentioned the existence of surveillance policies, protocols, or guidelines shared with reporting sites [15, 32, 33, 35, 37, 41–43]. Instead of a protocol document, Germany’s syndromic surveillance system team developed a toolkit hosted on an institutional website [17]**.** Some protocols were described as insufficient or poorly defined. For example, interviewees in several EU countries described their guidance on data collection and recording as inadequate [28]. Similarly, prior to the binational surveillance initiative, reporting protocols for US-Mexico land borders were described as poorly defined [41].

Policies related to surveillance were rarely mentioned. Only one source reported a surveillance-related policy change, in which the Italian surveillance system was extended beyond the humanitarian emergency end date to allow reporting centres to apply Italian infectious diseases statutory surveillance [15]. Conversely, Germinario et al. highlighted the EU’s need to enact infectious disease screening regulations for migrant populations [33]. A scoping study of six EU countries mentioned that “legal procedures” usually needed to be surpassed in destination countries, leading to ad-hoc activities [28].

### Reported lessons and limitations

Nine sources reported that migrant-specific surveillance systems provided insight into infectious diagnoses and trends among refugees, enabled early detection of potential outbreaks, helped reduce disease transmission in camps, and led to obvious improvements in public health interventions [17, 32, 33, 36–40, 42].

Three reported the additional benefit of closer collaboration between partners or with refugee populations. For example, the Albania source reported that setting up the surveillance system led to close collaboration between surveillance team and health facilities, a task that would have been difficult outside the emergency context [34]. Kouadio et al recounted a reason behind their effective surveillance was cooperation between the surveillance team and refugee population [38]. Waterman et al emphasised cross-border coordination for achieving surveillance goals and common guidelines between US and Mexico [41].

Many sources described lessons on limitations that needed to be addressed. For example, two WHO assessments showed data collection was not systematic, with different databases maintained by different partners, and health-workers in migrant centres noting aspects of the syndromic surveillance system needed to be clarified [19, 21]. Similarly, another mentioned the lack of systematic approaches to surveillance, especially in the EU [30]. One noted that in Greece some diseases were identified through the mandatory notification system operating in parallel to the Points of Care surveillance system for refugees and migrants [18]. Another limitation mentioned was sustainability of surveillance for refugee populations. A study of six EU countries declared sustainability as almost impossible, resulting in ad-hoc systems [28]. Another source from Italy similarly highlighted that ad hoc surveillance cannot be sustained so needs to be corrected “before it can become a routine tool” [15].

Napoli et al mentioned use of paper-based methods for reporting as a logistical limitation, because it was time consuming, led to less timeliness of reporting, and was one of the issues affecting sustainability of the system [15]. They advised shifting to an online reporting platform [15]. Riccardo et al, corroborated this observation that such logistical challenges contributed to underreporting [30].

## Discussion

This scoping review found minimal literature on disease surveillance for refugees in destination countries, with only 20 eligible sources included. Sources highlighted no systematic means of surveillance across migrant-receiving countries [19, 21, 30]. Few mentioned the existence of surveillance protocols and guidance necessary for accurate implementation and data sharing, while others described guidance as poorly defined or insufficient [28, 41]. Information on relevant policies shaping surveillance structures or activities was almost non-existent. Literature outside the European region appears very limited, despite significant migrant populations in the Eastern Mediterranean and North Africa since the Syrian conflict began [44].

During the significant and rapid increases in migration from war-devastated countries to Europe, ECDC emphasised the importance of establishing syndromic surveillance for refugee populations as a complement to routine national surveillance systems. Despite the presence of EU laws requiring countries to report infectious diseases, these systems have not been particularly effective [43]. In 2016, ECDC published guidance for countries on establishing their own syndromic surveillance to more quickly detect, investigate, and respond to potential epidemics in countries receiving refugees [13, 14]. This guidance and advocacy on establishing surveillance in the EU may help explain why most surveillance literature for refugees described European experiences.

Italy had the most sources on this topic and has been described by the ECDC as having an exemplary syndromic surveillance system [13, 14]. Sources describing the Italian experience provided relevant lessons about strengths and weaknesses of ad hoc surveillance in terms of fluidity of migrant populations, migration centres’ compliance, and lack of sustainability due to time and resource constraints [15]. We would encourage more countries hosting refugees to publish their surveillance experiences to help all countries identify surveillance methods, outcomes, and challenges to guide surveillance activities in future refugee responses.

Most literature on migrants and refugees focused on screening rather than surveillance systems. In some instances, infectious diseases among refugees underreported through surveillance were identified through screening [43, 45]. Rossi et al found that infectious disease reporting accuracy for migrants might be limited by their healthcare access, which other authors have reported as being restricted or challenging, though they also noted possible reporting inaccuracies in screening [17, 22, 28, 43]. This suggests disease surveillance for refugees could potentially be more effective in combination with health screening.

Another important finding in this review is the role of international organizations in surveillance activities. Our review found that UNHCR and WHO either implement new surveillance systems or support existing ones depending on country/border situations. In the absence of official surveillance, WHO or UNHCR have led on establishing surveillance systems [35, 36, 39]. Such international involvement in surveillance stems from organisational mandates to protect health globally and across borders [46, 47]. An example is WHO’s Global Outbreak and Response Network (GOARN) that deploys technical assistance to areas of need [46, 47].

Several limitations should be considered. First, scoping reviews only include sources within authors’ search capacity (e.g. accessible on databases searched or through stakeholders). Based on the authors experience in surveillance, it is likely that much of the work on this topic remains unpublished since routine activities are often not considered sufficiently interesting to publish and during humanitarian emergencies publishing may not be a priority. Further, some research might be inaccessible (e.g. due to numerous potential search terms, political sensitivities, and in many countries refugee surveillance is integrated within routine national surveillance systems). Second, this review focused on infectious diseases generally and did not include sources only describing tuberculosis and HIV surveillance. As these are the most frequently screened diseases, due to their importance and possibly the abundance of dedicated guidelines, and since a scoping review of tuberculosis screening and surveillance was recently conducted, we considered it more useful to include infectious diseases more broadly [43, 48]. Third, we did not include sources without an English abstract so useful non-English sources may have been missed [49]. Fourth, we did not include infectious disease surveillance for refugees targeting SARS-CoV-2, since we conducted this review before the start of the COVID-19 pandemic. Despite a plethora of literature on COVID-19 surveillance, this is limited when addressing vulnerable at risk populations such as refugees [50–52]. It would be informative to have a specified review in the future addressing implementation of surveillance activities for refugees during pandemics, in particular COVID-19. Finally, we did not assess evidence quality, as the quantity and quality of sources were insufficient to do this meaningfully.

## Conclusion

This scoping review examines the scope of the literature on infectious disease surveillance for refugees at borders and in destination countries. Though this review was conducted up to July 2021, only 20 sources were found between 1999 and 2019. Surveillance systems for refugees were primarily syndromic, countries were primarily European, and little was documented on surveillance policies or protocols. Further documentation is needed to address gaps in this literature and help guide countries welcoming refugees to set up or enhance infectious disease surveillance systems accordingly.

## Data Availability

Data sharing is not applicable to this article, all articles used for the review are in the references section.

## Abbreviations

CARE: Common Approach for Refugees
US-CDC: Centers for Disease Control (USA)
ECDC: European Centres for Disease Control
EMBASE: Excerpta Medica Database
EU: European Union
EURO: European Regional Office (WHO)
EWAR: Early Warning and Response
IOM: International Organization for Migration
MESH: Medical Subject Headings
PRISMA: Preferred Reporting Items for Systematic Reviews and Meta-Analyses
UNHCR: United Nations Refugee Agency
WHO: World Health Organization
